# Parents’ Perceived Barriers to Accessing Sports and Recreation Facilities in Ontario, Canada: Exploring the Relationships between Income, Neighbourhood Deprivation, and Community

**DOI:** 10.3390/ijerph14101272

**Published:** 2017-10-23

**Authors:** Daniel W. Harrington, Jocelyn W. Jarvis, Heather Manson

**Affiliations:** Health Promotion, Chronic Disease and Injury Prevention, Public Health Ontario, 480 University Ave., Toronto, ON M5G 1V2, Canada; jocelynjarvis0@gmail.com (J.W.J.); heather.manson@oahpp.ca (H.M.)

**Keywords:** access to sports and recreation facilities, physical activity, socioeconomic status, income, neighbourhood deprivation, parents

## Abstract

Sports and recreation facilities provide places where children can be physically active. Previous research has shown that availability is often worse in lower-socioeconomic status (SES) areas, yet others have found inverse relationships, no relationships, or mixed findings. Since children’s health behaviours are influenced by their parents, it is important to understand parents’ perceived barriers to accessing sports and recreation facilities. Data from computer assisted telephone interviews with parents living in Ontario, Canada were merged via postal codes with neighbourhood deprivation data. Multivariable logistic regression modeling was used to estimate the likelihood that parents reported barriers to accessing local sports and recreation facilities. Parents with lower household incomes were more likely to report barriers to access. For each unit increase in deprivation score (i.e., more deprived), the likelihood of reporting a barrier increased 16% (95% CI: 1.04, 1.28). For parents, the relationships between household income, neighbourhood-level deprivation, and barriers are complex. Understanding these relationships is important for research, policy and planning, as parental barriers to opportunities for physical activity have implications for child health behaviours, and ultimately childhood overweight and obesity.

## 1. Introduction

The benefits of physical activity for health, chronic disease prevention and premature mortality have been well-established [1,2,3]. Among other health behaviours, the places where we live, work, play, and interact with others can have an overarching influence on physical activity, and ultimately contribute to rates of overweight, obesity, and cardiovascular disease [4,5,6,7,8]. Facilities for sports and recreation (e.g., fitness centres, recreation centres, tennis courts, basketball courts, parks and playgrounds) can facilitate public health by providing valuable places for engaging in physical activities [9]. Availability of these facilities has been shown to increase engagement in physical activities from walking for leisure to more vigorous activities across many population groups (e.g., children, youth and adolescents, adults and older adults) [10,11,12,13,14,15,16,17,18,19,20,21]. As such, common composite indices and methods for characterizing healthy neighbourhoods generally include some measure of availability of facilities for physical activity [3,5,22].

A considerable amount of research attention has been given to understanding the relationships between availability and social determinants of health, particularly by looking at spatial distributions of sports and/or recreation facilities. The majority of this work has focused on the relationship between facility availability and socioeconomic status (SES). The evidence supporting this relationship is mixed, and dependent on several factors including the type of facility [23]. Certainly, there is evidence to suggest that availability of facilities is worse for those living in lower-SES areas [24,25,26,27,28,29,30,31,32,33], indicating that a group already at risk for lower levels of physical activity [31] are also disadvantaged in terms of their opportunities for physical activity. Others have found the inverse relationship between availability and SES [34,35,36,37], though car ownership in higher SES areas has been found to mitigate this disadvantage [35]. Some studies have reported mixed findings, whereby publicly-funded sports and recreation facilities are more available to those living in lower SES, while privately-funded facilities are more available to those in higher SES areas [38,39,40]. This research raises questions about differences in the quality of facilities, and the extent to which ‘good’ or ‘useable’ facilities are available in lower SES areas [37,41]. Arbel and colleagues have also shown that it can be more expensive to participate in facility-based physical activities in lower SES areas [42]. Other studies have found no relationship between availability and area-level SES [12,23,30,43,44]. To a lesser degree, area-level ethnic diversity and degree of rurality have been explored as determinants of the availability of sports and recreation facilities [27,28,32,34,45,46,47,48]. There are two clear indications from this literature. The first is that where you live influences the physical availability of facilities. The second is that understanding these relationships requires more research.

Borrowing from the health services literature, we know that availability is a necessary, but insufficient measure of access [49]. Applied in the context of this research, availability of sports and recreation facilities are a measure of potential access rather than realized access [50], which considers barriers at individual, interpersonal, and environmental levels in addition to availability [49]. Barriers to using sports and recreation can take many forms, and may be related to ability, gender, culture, socioeconomic circumstance, facility-related factors, communication, or environment [51]. Specific to using sports and recreation facilities, (perceived) quality of the facility, as mentioned previously, or limited programming offered by the facility have been raised as potential barriers to access, even in cases where objectively measured availability is considered to be adequate [37,41]. 

In Canada, as in many industrialized countries, childhood overweight and obesity represent a significant public health issue. Most current Canadian estimates indicate that based on z-scores for objectively measured Body Mass Index, 27% of children aged 3–19 are affected by overweight or obesity [52]. Further, only 9% of boys and 4% of girls are meeting current Canadian physical activity guidelines—60 min per day of moderate-to-vigorous physical activity [53]. Access to sports and recreation facilities is important for children’s physical activity [30,54], and especially so with children being unable to meet recommended levels through schools only [30]. To effectively address childhood overweight and obesity through increasing physical activity, it is important to understand the context in which children access these facilities. Applying a socio-ecological lens [55,56], children’s health behaviours can be conceptualized as being determined by interactions between individual factors (i.e., intrapersonal), but also parental and peer support (i.e., interpersonal), family and environmental factors (i.e., structural) [57,58]. With respect to parental influence, for example, a recent study found that taking children to places where they can be physically active increases the likelihood that children reach guidelines for daily moderate to vigorous physical activity [59].

Parents’ perceptions can affect children’s health and health behaviours [60]. As gate-keepers of children’s health behaviours [59] it is important to understand parents’ perceived barriers to accessing opportunities for physical activity. Compared to some groups such as low income individuals [51,61,62,63] and those with disabilities [64], barriers to accessing sports and recreation facilities for parents is understood less. In the studies that have considered parent-reported barriers, some have reported perceived limited availability and access to facilities as barriers to use [29,54,65,66]. Others have reported perceived safety due to crime [54], perceived poor quality of the facility [30], lack of programming and accommodations [30,66] and lack of culturally appropriate programming [67] as barriers to use. A qualitative study of parents’ barriers, Thompson and colleagues identified busy lifestyles, age of children, weather, lack of transportation and costs as key barriers to using sports and recreation facilities [65].

Previous research has generally focused on local studies, limiting exploration about variation in access across different communities. As well, little is known about the relative roles of household income and neighbourhood deprivation, or about the types of barriers that parents are frequently faced with. To address these gaps, this study uses data from a survey of parents and guardians of children under the age of 18 living in Ontario, Canada to: (1) contribute to understanding the extent to which household income, neighbourhood deprivation and community of residence to parents’ perceived barriers to access; (2) explore common types of barriers to access faced by parents; and (3) examine if different types of parents experience different types of barriers.

## 2. Materials and Methods

Data for this study were collected by Public Health Ontario using computer assisted telephone interviews between February and March 2015. The survey was developed for the purpose of evaluating a provincial community-based program targeting childhood overweight and obesity in Ontario, Canada. This study is a secondary analysis of these baseline data, which were collected prior to the start of implementation of this program. Specifically, the target population for the survey was parents of children under the age of 18, living in Ontario. Parents were contacted using both landlines and mobile phones using a random digit dialing approach. No incentives were offered, and active consent was obtained for each respondent. In the case of two-parent households, the parent with the most knowledge about their child’s health was asked to respond. The survey primarily focused on parent support for child health behaviours (i.e., physical activity, healthy eating, screen time reduction, and sleep). However, the respondents were also asked about their perceptions of the community that they lived in, including perceived access to sports and recreation facilities. Demographic information was collected from all parents that completed the survey. Respondents were also asked to provide their postal code.

The survey achieved a cooperation rate of 12% (Calculated as the number of people who participated, divided by the number of eligible people with whom contact was made.) based on American Association for Public Opinion Research standard definitions [68]. Of the 3206 respondents, more than 20% (*n* = 677) did not report their household income. Missing cases were imputed using regression predictions [69] based on parent age, marital status, home ownership, highest level of completed education, and time since immigration. For each predicted income value, random prediction error was added by generating random deviates based on a normal distribution around the predicted value (i.e., mean) with a standard deviation equal to the residual standard error of the fitted model. This process resulted in successful imputation for 591 or the 677 missing cases (87.3%). In cases where the variables used to build the regression prediction model were also missing, the missing income cases remained as missing.

To assess potential response bias, parents in the sample were compared to a sub-sample of a provincially representative health survey (i.e., the Canadian Community Health Survey (CCHS)). The sub-sample is representative of age-sex strata in the province, and is not necessarily representative of parents in Ontario; however, it provides a reasonable comparison. Table 1 presents a comparison between the study sample and the sub-sample from the CCHS. In general, those in the study sample were more likely to be female (possibly because female parents are most knowledgeable about their child’s health), Canadian-born, have a post-secondary education, have a higher income, and be from a lone-parent family.

The primary outcome variable explored in this study is derived from an open-ended question parents answered following a battery of questions about their community. As part of these questions, parents were asked to respond on a five-point Likert scale on their agreement with the following statements: (1) *In my community, there are good facilities for sports and recreation available*; and (2) *My family uses the sports and recreation facilities in my community*. Parents were then asked about the reasons why their family may not be able to use these facilities (i.e., barriers). This item was asked as an open-ended question and parents could provide multiple responses. Parents coded as having no barriers must have explicitly reported that there was nothing that made accessing sports and recreation facilities difficult for their family. Some coding categories were pre-identified, and interviewers could indicate these responses if they were given. If a response was outside of the pre-identified barriers, it was coded as ‘Other’ and recorded verbatim.

Two hundred and eight of the responses were open-ended (i.e., coded as ‘Other’). Two coders independently coded 25% of the verbatim responses in order to identify new coding categories, or to code any responses that fit into the pre-identified categories. At this stage, the coders then clarified any differences in new coding categories. The coders then coded an additional 25% of the open-ended responses with the agreed-upon codes. Cohen’s kappa was calculated to establish inter-rater reliability, and exceeded the acceptable agreement (*k* = 0.71), so a single coder completed the remainder of the coding. This approach to coding has previously been used to code barriers to supporting child health behaviours, collected in a similar manner [70]. Given the granularity with which the barriers were coded through this process, individual barriers were aggregated up to one of four barrier types for analysis, informed by the socio-ecological perspective: child-level, parent-level, facility-related, and environmental (Table 2).

To assess the role of neighbourhood-level deprivation, data from the survey were linked via parent-reported postal code to dissemination areas (DAs) using Statistics Canada’s 2015 Postal Code Conversion File (PCCF). DAs are the smallest standard administrative boundary available in Canada, and represent populations of 400 to 700 individuals [71]. DAs are used as proxy measures for neighbourhood in this study. Of the 3206 respondents to the survey, 51 did not provide a postal code. An additional 95 were unable to be linked to a DA through the PCCF.

Data were subsequently linked at the DA level to Ontario Marginalization index (ON-Marg) data [72]. The most recent ON-Marg data are a provincial subset of the Canadian Marginalization Index (CAN-Marg), which is an index based on the 2006 Canadian census [73]. The purpose of ON-Marg is to measure differences in marginalization between geographies as measured by four key dimensions: residential instability, material deprivation, ethnic concentration, and dependency. For this study, we focus on the material deprivation dimension at the DA-level, calculated based on the following indicators: proportion of the population aged 20+ years without a high school diploma; proportion of families who are lone-parent families; proportion of the population receiving government transfer payments; proportion of the population aged 15+ years who are unemployed; proportion of the population considered low-income; and, proportion of households living in dwellings that are in need of major repair.

The details of how CAN-Marg indices are constructed are available elsewhere [73]. Briefly, factor scores of material deprivation are constructed from a principal component factor analysis. Lower scores correspond to areas that are the least deprived, and higher scores correspond to areas that are most deprived. At the provincial level, marginalization scores are also grouped into quintiles, with Q1 representing the least deprived areas.

It should be noted that at the DA level, some census data can be suppressed by Statistics Canada to protect the privacy of individuals, limiting the number of cases that could be linked. Of the 3060 respondents that could possibly be assigned a DA based on their reported postal code, 2661 were successfully merged with ON-Marg data (83.0% of the original sample). Analyses for this study were based on the subsample of 2661 parents for whom neighbourhood-level ON-Marg data were linked.

All analyses were conducted using R v. 3.2.3. Prior to undertaking the analysis, bivariate analysis was used to compare the subsample (*n* = 2661) with the sample that was unable to be linked to ON-Marg data (*n* = 545).

In order to understand the relative contributions of household income, neighbourhood deprivation, and community of residence to parents’ barriers to accessing sports and recreation facilities, a cross-classified multilevel logistic model was built. The outcome variable was a binary variable indicating whether or not the parent reported a barrier to accessing sports and recreation facilities. Independent variables at the individual level included: parent age, parent gender, marital status (‘married’ or ‘common law’ versus ‘other’), time since immigration (‘Canadian-born’, ‘0–9 years’, ‘10 or more years’), parent education (‘Less than secondary’, ‘Secondary’, ‘Post-secondary’), household income (‘Less than $30,000’, ‘$30,000–59,999’, ‘$60,000–99,999’, ‘More than $100,000’), and area size based on DA of residence (‘Rural’, ‘Small/Medium’, ‘Large urban’). Data on area size are available in the PCCF file. ‘Small/Medium’ areas are population centres (population of at least 1000 and population density of at least 400 persons per square kilometre) with 1000–99,999 residents, ‘Large urban’ areas are population centres with 100,000 or greater residents, ‘Rural’ areas do not meet the criteria for population centres. Area size was treated as an individual-level variable.

Two second-level grouping variables were used initially: (1) neighbourhood deprivation quintile (*n* = 5); and (2) community of residence, defined by census sub-division (*n* = 250). Census sub-divisions are the administrative boundaries associated with municipalities in Canada [74]. In comparison with the null model containing only the intercept, there was no significant improvement of model fit (based on deviance) by allowing the intercept to vary randomly across community of residence (Intraclass Correlation Coefficient (ICC) = 0.0035). There was significant model improvement over the null model allowing the intercept to vary randomly across deprivation quintiles (*p* < 0.05); however, the proportion of the variation in the outcome explained by deprivation quintile was small (ICC = 0.001).

ON-Marg indices can be used as individual-level measures of deprivation when appropriate [75]. Given the lack of clustering of individuals within neighbourhoods (i.e., DAs) in this study, the raw deprivation scores (versus quintiles) were included as an additional independent variable at the individual-level to compare with the multilevel model where intercepts varied across deprivation quintiles. Model fit statistics indicated that the former model fit the data better than the multilevel model. Given this, and the fact that there was no significant variation across communities, the cross-classified multilevel logistic model was abandoned for a multivariable logistic regression.

Coefficients from the logistic regression are presented in the regression as odds ratios (ORs) with 95% confidence intervals (CIs). ORs can be interpreted as the odds that a respondent reported a barrier to accessing sports and recreation facilities relative to the reference category of the variable, adjusted for all other variables in the model. Marital status and time since immigration did not significantly improve model fit, and were removed from the final model. As well, though child age was available in the data set, it was not found to be significant to the model, and given the high correlation with parent age, it was removed from the model. Parent age remained in the model as a potential predictor of the perception of barriers. Subsequent to fitting the regression model, bivariate chi-square tests were conducted to better understand the types of barriers that may be faced by different demographic or socioeconomic groups. While we desired to fit multivariable models for each of the types of barriers, this analysis was limited by small sample sizes for child-level barriers. Given that we wished to analyze each of the barrier types similarly, we limited the analysis to bivariate relationships.

## 3. Results

A description of the subsample versus those who could not be linked with ON-Marg is presented in Table 3. There were no significant differences between the groups in terms of the likelihood that they reported a barrier (48.3% in the subsample versus 48.5%), or in the average number of barriers reported (0.66 in the subsample versus 0.68). There were no significant differences in parent’s age or gender between the two groups. Parents in the subsample were significantly more likely to have a partner (88.2% versus 84.7%), have a post-secondary education (88.5% versus 83.9%), and have higher household incomes. Parents in the subsample were less likely to be Canadian-born (64.7% versus 78.8%), and less likely to be from large urban areas (64.0% versus 86.6%). The average deprivation score for parents in the subsample was −0.211 (standard deviation 0.87), and in general, they were more likely to be in the first three neighbourhood-level deprivation quintiles (less deprived) than in Q4 or Q5 (more deprived).

Overall, most parents agreed that there were good facilities for sports and recreation available in their community (83.5% agree/strongly agree), and reported that their family used the facilities (77.0% agree/strongly agree). Parents who reported that there were good facilities available in their community were significantly less likely to report any barriers to using the facilities (6.1%) compared with those who did not (28.0%; Chi-square = 216.8, *p* < 0.05). A Wilcoxon rank-sum test showed that on average, these parents also reported significantly fewer barriers (0.59) than others (1.1; *p* < 0.05). Likewise, parents that reported that their family used the facilities in their community were less likely to report any barriers compared to those that did not (11.2% versus 36.0%; Chi-square = 219.4, *p* < 0.05). These parents also reported significantly fewer barriers on average (0.55 versus 1.1; *p* < 0.05).

### 3.1. Regression Model

Table 4 presents the logistic regression model predicting the likelihood that parents reported barriers to using the sports and recreation facilities in their community. Female parents were almost 50 percent more likely than male parents to report barriers [OR: 1.47, 95% CI: (1.22, 1.77)]. With each increase in one year of parents’ age, there was a small but significant increase in the likelihood that they reported barriers [95% CI: (1.00, 1.02)]. There were no significant differences between parents who had a partner (i.e., married or common-law married) and those that did not. Relative to parents with a post-secondary education, those with less than secondary school were significantly less likely to report barriers [OR: 0.60, 95% CI: (0.37, 0.98)]. Parents living in rural areas were more likely to report barriers, compared to those in large urban areas [OR: 1.46, 95% CI: (1.17, 1.81)]. Compared to those in the highest household income category (More than $100,000), parents in other household income categories were more likely to report barriers, and particularly so for those in the lowest category (Less than $30,000) who were more than two and one-half times more likely to report barriers [OR: 2.61, 95% CI: (1.85, 3.69)]. Finally, after adjusting for all other variables in the model, the estimate associated with neighbourhood-level deprivation indicates that for each increase by one in ON-Marg deprivation score (indicating more deprivation), the likelihood that a parent reported a barrier increased by 16 percent (95% CI: 1.04, 1.28).

### 3.2. Types of Barriers

Overall, parent-level barriers to accessing sports and recreation facilities (63.6%) were most frequently reported, followed by facility-related barriers (17.8%), environmental barriers (14.9%) and child-level barriers (5.2%) (Table 5). Males were more likely to report parent-level barriers (72.4%) compared to females (62.9%), while females were more likely to report facility-related (21.0%) and environmental barriers (17.3%) than males (11.9% and 13.6%, respectively). Those living in rural areas were least likely to report parent-level barriers (56.2% versus 68.9%), and most likely to report environmental barriers (29.5%) compared to those in small/medium (11.9%) or large urban population centres (13.6%). Those in small/medium centres were more likely to report facility-related barriers (26.1%) compared with parents from other area sizes. There were no significant differences by level of education; however, parents in the highest household income group were more likely to report parent-level barriers (73.5%), while those in lower household income groups were more likely to report facility-related and environmental barriers. Finally, parents in the most deprived areas (Q5) were less likely to report parent-level barriers (55.4%) compared parents from other quintiles (Table 5).

## 4. Discussion

This study fills an identified gap in the literature by focusing on parents’ barriers to accessing sports and recreation facilities that simultaneously attempts to tease apart the influence of household income, neighbourhood marginalization, and community of residence. While the analytic plan for this research required some adjustment from the original design, this study has made a significant contribution to the literature on access to opportunities for physical activity (i.e., sports and recreation facilities) by focusing on parents as a unique population. First, when parents perceive barriers, they are also less likely to think that there are good facilities available in their community, and they are less likely to report that their family uses the facilities. This is an important take home message from this research with implications for public health. Other research has highlighted the importance of properly addressing perceived barriers to increase family engagement in physical activity [65]. In particular, the results of this study imply that interventions that acknowledge and accommodate parents’ perceived barriers to accessing sports and recreation facilities could have a positive impact on family and child physical activity.

We have also shown that, overall, parents (perhaps unfairly) took responsibility for the reasons why they experience difficulties accessing these facilities. More than 60% of parents cited parent-level barriers in comparison with child, facility, or environmental barriers (each less than 20%). Our interpretation of this is that parents’ competing priorities and busy schedules are “top-of-mind” when parents think about their barriers to access. This research provides yet another example of how environmental changes that help to make healthy choices easy—a fundamental principle of health promotion and public health outlined by the Ottawa Charter on Health Promotion more than 30 years ago [76]—could change how parents think about barriers to access, or help to accommodate their complex schedules. Additionally, there may be a role for family-level interventions that focus on scheduling, time management, or increasing awareness of alternative opportunities for physical activity.

Related to the main objective of this paper, the regression model showed that barriers were not experienced equally across household income or levels of neighbourhood-level deprivation. Overall, parents with lower levels of income were much more likely to report barriers to accessing sports and recreation facilities. The largest difference indicates that parents from households that earn less than $30,000 (CAD) annually were much more likely to report barriers than those from households that earn more than $100,000 (CAD). This finding echoes the findings from others who have found that low-income children have less access to facilities for physical activity [62], and that this is primarily due to financial barriers [61,62,65]. This point is further explored during discussion of the analysis of types of barriers.

There was a significant relationship with neighbourhood-level deprivation as measured by ON-Marg, after adjusting for household income. As deprivation increased, parents were more likely to report barriers to access. Indeed, neighbourhood income matters for access to facilities [30], and our results highlight the overarching influence that can be exerted across all household-level socioeconomic strata. This suggests that interventions aiming to improve access to opportunities for physical activity may require a two-pronged approach that prioritizes high-risk strategies in places that are less deprived, and population-level strategies in places that are most deprived.

Bivariate analyses of types of barriers shed further light on how particular groups of parents were differentially affected by different barriers. Male parents, for example, were more likely to report parent-level barriers, while female parents were more likely to indicate facility-related and environmental barriers. It may be that female parents are more likely to be the parent responsible for gaining access to facilities for the family, and are thus more sensitive to issues around programming and facility quality, or availability and safety at the environmental level. However, to comment definitively on this relationship would require more research using different sources of data, and different approaches, including qualitative ways of knowing.

Intuitively, it makes sense that parents in rural areas were more likely to perceive environmental barriers to access, given that availability of facilities in rural areas are known to be poor compared to urban areas [45]. It is interesting that those in small-medium urban areas were most likely to report facility-level barriers. This finding raises questions about the cost, quality, and programming available in facilities in these areas, which may have adequate availability, at least relative to rural areas.

In terms of household income the general indication is that parents from lower income households report more facility and environmental barriers, while parents from higher income households report more parent-level barriers. Though the relationships with facility-related and environmental barriers were not significant, a similar trend was evident across neighbourhood deprivation quintiles. The same significant relationship with parent-level barriers was apparent across neighbourhood deprivation. We surmise that those who are the least deprived at the household and neighbourhood levels may have better *availability* of facilities, so they identify themselves as the primary reasons why they have difficulties with access (parent-level barriers). However, at lower levels of household income, families are more likely to experience barriers related to cost, facility quality, program offerings and capacity, and availability of sports and recreation facilities.

This study has some limitations that highlight directions for future research. First, the cross-classified model specified in the original analytic plan was reduced to a multivariable logistic regression model. We maintain that the multilevel model was appropriate given the data, and encourage others to consider the complex ways in which their data are structured. However, there was no significant variation in barriers detected at the community-level as defined by census sub-division, limiting conclusions related to place of residence. We do not take this to mean that the community you live in does not matter for access to sports and recreation facilities. Rather, we believe this is a by-product of the modifiable areal unit problem [77], reflecting a limitation of how community boundaries were defined. A different, perhaps smaller, boundary may have been more appropriate (e.g., neighbourhoods); however, the data would not support this type of analysis given the large sample size of individuals that would be necessary. Third, objectively-defined availability (i.e., potential access) of facilities was not considered in this study. While we understand perceived barriers to shape behaviours *despite availability*, it would be interesting to introduce the ‘supply’ side of the equation to this analysis. Fourth, these data are not linked directly to health behaviour data for parents or children. In particular we think it would be an important next step to explore the moderating effect that barriers have on the relationship between access to facilities and physical activity. Finally, 12% of the parents reached on the phone participated in the survey, driven lower by mobile phone users (8% participation). As well, from the comparison with parents in the Canadian Community Health Survey, we know that parents who participated were more likely to be female, Canadian-born, and have higher levels of education and income. We note that interviewers asked to speak with the person most knowledgeable about the child’s health, and also that we only collected information in English and French, and only by telephone. These approaches likely introduced some sampling bias, and as such, the results presented in the paper should be considered in the context of these differences.

## 5. Conclusions

Despite its limitations, this paper makes some important contributions to the literature on access to opportunities for physical activity; in particular by focusing on a unique population (i.e., parents of children under the age of 18). Specifically, this study is one of the first to look at issues of access faced by parents across a large geography (i.e., the province of Ontario), and consider variation in access between communities. While community of residence was not significant in this analysis, parents living in different *types* of communities (e.g., rural, more deprived) were found to face barriers more often than others, and face different types of barriers. Understanding these relationships is important for research, policy and planning, as parental barriers to opportunities for physical activity have implications for adult, family, and child health outcomes. This is particularly important given the important role parents have to play in their children’s health, and the current low rates of participation in physical activity in childhood in Canada and other countries. In addition to addressing the perceived high costs of accessing sports and recreation facilities (e.g., by subsidizing associated costs; offering family discounts), a focus on creating supportive environments that make access easier (e.g., through more locations and/or a broader variety of programming) and more feasible (e.g., in consideration of the other constraints on parents’ time) may be particularly helpful for improving access to sports and recreation facilities for all parents and children.

## Figures and Tables

**Table 1:** Comparison of study sample with parents in CCHS (2013–2014).

Characteristic	Study Sample %	CCHS (2013–2014) %
Female	71.0	53.7
Partner	85.3	89.0
Immigration Status		
Canadian-born	76.5	59.9
0–9 years	6.6	11.4
10 or more years	16.9	24.1
Education		
Post-secondary	84.7	74.0
Secondary or Less	15.3	26.0
Household Income		
<$20,000	4.6	4.7
$20,000–39,999	8.4	13.2
$40,000–59,999	12.2	14.1
$60,000–79,999	13.6	13.2
$80,000–99,999	15.5	12.5
>$100,000	45.3	42.3

**Table 2:** Types of barriers to accessing sports and recreation facilities.

Child Barriers	Parent Barriers	Facility-Related Barriers	Environmental Barriers
Child’s age (i.e., too young) Child’s health (e.g., disabilities, other health issues) Child preferences (e.g., doesn’t like sports; prefers art programs)	Lack of time/busy Work schedule Inconvenience Lack of parent interest Transportation issues not related to public transportation Lack of parent knowledge or skills (e.g., do not know how to gain access) New to community Lone parent	Cost Programming issues (e.g., lack of programs for child’s age; type of programming offered) Overcrowded/busy Poor facility quality (e.g., outdated, old, not accessible for people with disabilities) Poor program capacity (e.g., always full, cannot get child a spot in a program) Hours of operation Lack of advertising Membership required Staffing issues (e.g., not enough staff; poorly trained staff)	Distance No facilities available (or, not enough facilities) Lack of public transportation Weather (e.g., closed in the winter) Safety (e.g., lack of supervision; unsafe)

**Table 3:** Comparison of linked subsample with unlinked parents.

Characteristic	Subsample (*n* = 2661) %	Unlinked (*n* = 545) %
Reported any barriers	48.3	48.5
Barriers	Mean: 0.66	Mean: 0.68
Female	67.7	71.7
Age	Mean: 42.0	Mean: 42.0
Partner *	88.2	84.7
Immigration Status *		
Canadian-born	64.7	78.8
0–9 years	11.1	5.7
10 or more years	24.2	15.5
Education *		
Post-secondary	88.5	83.9
Secondary	9.6	13.0
Less than Secondary	1.9	3.1
Household Income *		
<$30,000	7.4	9.7
$30,000–59,999	13.0	16.7
$60,000–99,999	30.0	28.8
>$100,000	49.6	44.7
Area Size *		
Large Urban	64.0	86.6
Small/Med. Urban	17.6	9.0
Rural	18.4	4.4
Deprivation Quintile		
Q1 (Least deprived)	24.4	-
Q2	24.5	-
Q3	22.4	-
Q4	15.6	-
Q5 (Most deprived)	13.1	-

* Indicates significant differences in proportions.

**Table 4:** Logistic regression predicting likelihood of reporting barriers to sports and recreation facilities.

Independent Variable	Unadjusted Odds Ratio (95% CI)	Adjusted Odds Ratio (95% CI)
Gender (Ref: Male)		
Female	1.42 (1.22, 1.67)	1.47 (1.22, 1.77)
Age	1.00 (0.99, 1.01)	1.01 (1.00, 1.02)
Marital Status (Ref: No partner)		
Partner	0.72 (0.59, 0.88)	1.06 (0.83, 1.37)
Education (Ref: Post-secondary)		
Less than Secondary	0.95 (0.63, 1.45)	0.60 (0.37, 0.98)
Secondary	1.19 (0.96, 1.49)	0.97 (0.75, 1.25)
Area size (Ref: Large Urban)		
Rural	1.38 (1.13, 1.69)	1.46 (1.17, 1.81)
Small/Medium	1.11 (0.91, 1.36)	1.05 (0.84, 1.31)
Household income (Ref: More than $100,000)		
Less than $30,000	2.30 (1.77, 3.00)	2.61 (1.85, 3.69)
$30,000–59,999	2.21 (1.79, 2.74)	2.40 (1.86, 3.10)
$60,000–99,999	1.39 (1.17, 1.65)	1.37 (1.13, 1.67)
DA deprivation score	1.24 (1.14, 1.36)	1.16 (1.04, 1.28)

**Table 5:** Bivariate analysis of types of barriers by parent characteristics.

Parent Characteristic	Child Barriers (%)	Parent Barriers (%)	Facility-Related Barriers (%)	Environmental Barriers (%)
Gender				
Male	5.4	**72.4**	**11.9**	**13.6**
Female	5.2	**62.9**	**21.0**	**17.3**
Education				
Less than secondary	10.0	63.8	17.5	15.0
Secondary	5.3	60.9	21.4	18.6
Post-Secondary	5.1	66.6	18.0	15.8
Area Size				
Rural	4.6	**56.2**	**14.3**	**29.5**
Small/Medium	4.8	**62.9**	**26.1**	**11.9**
Large	5.6	**68.9**	**17.7**	**13.6**
Household Income				
<$30,000	8.7	**52.3**	**30.3**	**22.0**
$30,000–59,999	7.3	**55.2**	**25.2**	**21.8**
$60,000–99,999	5.0	**63.5**	**18.5**	**17.4**
>$100,000	3.9	**73.5**	**13.9**	**12.0**
Deprivation Quintile				
Q1 (Least deprived)	4.4	**70.0**	15.7	16.0
Q2	3.9	**66.6**	19.8	16.9
Q3	5.1	**66.8**	17.3	14.7
Q4	6.7	**64.1**	18.3	17.1
Q5 (Most deprived)	8.0	**55.4**	22.9	17.0

Bold font indicates a significant relationship, *p* < 0.05.
